# Limited Efficacy of α-Conopeptides, Vc1.1 and RgIA, To Inhibit Sensory Neuron Ca_V_ Current^1^,^2^,^3^

**DOI:** 10.1523/ENEURO.0057-14.2015

**Published:** 2015-01-30

**Authors:** Andrew B. Wright, Yohei Norimatsu, J. Michael McIntosh, Keith S. Elmslie

**Affiliations:** 1 The Baker Laboratory of Pharmacology, Department of Pharmacology, Kirksville College of Osteopathic Medicine, at Still University of Health Sciences, Kirksville, Missouri 63501; 2 Department of Physiology, Kirksville College of Osteopathic Medicine, at Still University of Health Sciences, Kirksville, Missouri 63501; 3 George E. Wahlen Veterans Affairs Medical Center and Departments of Psychiatry and Biology, University of Utah, Salt Lake City, Utah 84148

**Keywords:** alpha9/alpha10 AChR current, analagesic mechanisms, baclofen, CaV2.2 current, rat sensory neurons

## Abstract

Chronic pain is very difficult to treat. Thus, novel analgesics are a critical area of research. Strong preclinical evidence supports the analgesic effects of α-conopeptides, Vc1.1 and RgIA, which block α9α10 nicotinic acetylcholine receptors (nAChRs). However, the analgesic mechanism is controversial. Some evidence supports the block of α9α10 nAChRs as an analgesic mechanism, while other evidence supports the inhibition of N-type Ca_V_ (Ca_V_2.2) current via activation of GABA_B_ receptors. Here, we reassess the effect of Vc1.1 and RgIA on Ca_V_ current in rat sensory neurons. Unlike the previous findings, we found highly variable effects among individual sensory neurons, but on average only minimal inhibition induced by Vc1.1, and no significant effect on the current by RgIA. We also investigated the potential involvement of GABA_B_ receptors in the Vc1.1-induced inhibition, and found no correlation between the size of Ca_V_ current inhibition induced by baclofen (GABA_B_ agonist) versus that induced by Vc1.1. Thus, GABA_B_ receptors are unlikely to mediate the Vc1.1-induced Ca_V_ current inhibition. Based on the present findings, Ca_V_ current inhibition in dorsal root ganglia is unlikely to be the predominant mechanism by which either Vc1.1 or RgIA induce analgesia.

## Abstract (precis)

Better analgesic drugs are desperately needed to help physicians to treat pain. While many preclinical studies support the analgesic effects of α-conopeptides, Vc1.1 and RgIA, the mechanism is controversial.

## Significance Statement

Better analgesic drugs are desperately needed to help physicians to treat pain. While many preclinical studies support the analgesic effects of α-conopeptides, Vc1.1 and RgIA, the mechanism is controversial. The development of improved α-conopeptide analgesics would be greatly facilitated by a complete understanding of the analgesic mechanism. However, we show that we cannot reproduce one of the proposed analgesic mechanisms, which is an irreversible inhibition of Ca_V_ current in a majority of sensory neurons.

## Introduction

Severe pain reduces the quality of life of millions of people each year (Cousins et al., 2004). Conventional treatment for chronic pain includes opiates and nonsteroidal anti-inflammatory agents. However, the therapeutic potential of these treatment options for chronic pain are often limited by the development of serious adverse effects and tolerance. Thus, the discovery of improved drug therapies is of great importance.

α-conopeptides are small, disulfide-rich peptides that are isolated from the venom of the *Conus* genus of carnivorous marine snails and that block nicotinic acetylcholine receptors (nAChRs) (McIntosh et al., 2009). Two α-conopeptides, Vc1.1 and RgIA, have been shown to display antinociceptive effects in animal models; however, the mechanism responsible for analgesia remains debated (Vincler et al., 2006; McIntosh et al., 2009; Napier et al., 2012). Early studies found these α-conopeptides to be potent antagonists of heterologously expressed and native α9α10 nAChRs (Ellison et al., 2006; Vincler et al., 2006). Other studies have found that Vc1.1, but not RgIA, also weakly antagonizes nAChRs subtypes expressed in the periphery containing the α3 subunit (Clark et al., 2006; Ellison et al., 2006). Analogs of Vc1.1 that retain their specificity for α9α10 nAChRs, but not nAChRs with the α3 subunit, are devoid of analgesic effects in animal pain models (Nevin et al., 2007). These findings indicate the possible involvement of off-target effects being responsible for analgesia. However, mice lacking α9 nAChRs have reduced mechanical hyperalgesia in both neuropathic and inflammatory pain models, supporting a role for α9α10 nAChRs as a target for treatment of chronic pain (Mohammadi and Christie, 2014).

One group has proposed that the antinociceptive effects of Vc1.1 and RgIA are elicited by inhibition of N-type Ca_V_ (Ca_V_2.2) channels via activation of GABA_B_ receptors (Callaghan et al., 2008; Callaghan and Adams, 2010; Klimis et al., 2011; Adams et al., 2012; Mohammadi and Christie, 2014). The analgesic effects of GABA_B_ receptor activation by the specific GABA_B_ receptor agonist baclofen have been previously shown (Franek et al., 2004). Furthermore, GABA_B_ receptor activation inhibits the activity of N-type Ca_V_ channels (Ca_V_2.2) and inhibition of N-type channels expressed by nociceptors in the spinal cord dorsal horn is analgesic (Raingo et al., 2007). Pain relief comes from the reduction of excitatory neurotransmitter release (e.g., glutamate) from nociceptive nerve terminals when presynaptic N-channels are blocked (Elmslie, 2004; Miljanich, 2004; McIntosh et al., 2009). The inhibition of N-type Ca_V_ current by Vc1.1 and RgIA requires functional GABA_B_ receptors since the effect can be blocked by either application of a GABA_B_ receptor antagonist (Callaghan et al., 2008) or the knockdown of GABA_B_ receptors by siRNA (Cuny et al., 2012).

While inhibition of N-type Ca_V_ channel activity is a potential mechanism for Vc1.1- or RgIA-induced analgesia, this hypothesis is controversial (McIntosh et al., 2009). Neither Vc1.1 or RgIA were able to prevent the binding of a specific competitive antagonist to the human GABA_B_ receptor and both failed to activate GABA_B_ receptors expressed in *Xenopus laevis* oocytes (McIntosh et al., 2009). In addition, Vc1.1 failed to affect excitatory postsynaptic currents (eEPSCs) in the dorsal horn of rat spinal cord, which were almost completely blocked by baclofen (Napier et al., 2012). These findings are inconsistent with GABA_B_ receptor-induced inhibition of N-type Ca_V_ channels as the mechanism for analgesia produced by Vc1.1 and RgIA. Given these findings, there is a question of whether the Ca_V_ current inhibition in sensory neurons can be independently reproduced. The data presented here shows that the inhibition of Ca_V_ current in sensory neurons is on average either small (Vc1.1) or insignificant (RgIA), and that activation of GABA_B_ receptors is not consistent with the small inhibition induced by Vc1.1.

## Materials and Methods

### Animals

All animal procedures were performed in accordance with the authors' university animal care committee's regulations and were consistent with the National Research Council *Guide for the Care and Use of Laboratory Animals.* Adult male Sprague Dawley rats (200 − 400 g; Hilltop Lab Animals) were used in these experiments. The rats were housed in a U.S Department of Agriculture-approved, Association for Assessment and Accreditation of Laboratory Animal Care-certified animal care facility at a constant temperature 24 ± 1°C, under controlled 12:12 h light-dark cycles, and fed a standard rat chow diet and tap water *ad libitum*.

### Isolation of DRG neurons

The rats were euthanized by CO_2_ inhalation followed by decapitation using a laboratory guillotine (Kent Scientific) (Ramachandra et al., 2012). The lumbar 4 (L4) and L5 dorsal root ganglia (DRG) were isolated and dissociated in Earle’s balanced salt solution containing (in mg/ml): 0.7 collagenase, 1 trypsin, and 0.1 DNase at 37°C for 60 min (Ramachandra et al., 2012). The dissociated neurons were washed in minimum essential media (MEM) containing 10% fetal bovine serum (FBS) and plated onto polylysine-coated glass coverslips (Fisher Scientific). The isolated neurons were maintained overnight in a 5% CO_2_ incubator at 37°C in MEM supplemented with 10% FBS and 1% penicillin-streptomycin and used within 12 − 24 h (Ramachandra et al., 2012).

### Electrophysiological recordings from sensory neurons

The extracellular recording solution contained (in mM): 5 BaCl_2_, 145 NMG·Cl, 10 NMG·HEPES, and 15 glucose, with pH = 7.4 and osmolarity = 350 mOsm. The intracellular solution contained (in mM): 104 NMG·Cl, 14 creatine·PO_4_, 6 MgCl_2_, 10 NMG·HEPES, 5 Tris·ATP, 10 NMG_2_·EGTA, and 0.3 Tris_2_·GTP with pH = 7.4 and osmolarity = 335 mOsm. In some experiments, 0.1 mg/ml bovine serum albumen (BSA) was added to the external solution along with Vc1.1, but no enhancement of the Ca_V_ current inhibition was observed relative to Vc1.1 without BSA (same 5 neurons). Thus, the results combine conopeptide and baclofen data both with and without BSA.

Ionic currents were recorded using the whole-cell configuration of the patch-clamp technique with an Axopatch 200B amplifier (Molecular Devices) and digitized with an ITC-18 A/D converter (Instrutech Corp). Microelectrodes with a resistance of 2 − 5 MΩ were pulled from Schott 8250 glass (King Precision Glass) on a Sutter P-97 puller (Sutter Instruments). Series resistance was compensated by at least 80% using the electronic circuits of the Axopatch 200A amplifier. Neurons were voltage clamped at a holding potential of −80 mV and Ca_V_ currents were assessed using a three-step voltage protocol that tests for voltage-dependence of Ca_V_ channel inhibition (Elmslie et al., 1990; Ikeda, 1991; Ehrlich and Elmslie, 1995).

Experiments were controlled by a Power Macintosh computer (Apple Computer) running S5 data acquisition software written by Dr. Stephen Ikeda (NIH, NIAAA, Bethesda, MD). Leak current was subtracted from the step current using a −P/4 protocol. All experiments were conducted at room temperature (Ramachandra et al., 2012).

Data were analyzed with IgorPro (WaveMetrics) running on a Macintosh computer. Cell diameter was calculated from the cell capacitance as measured by the Axopatch circuitry, assuming a specific membrane capacitance of 1 µF/cm^2^ and that the neuron was spherical (Ramachandra et al., 2012).

### Preparation and microinjection of oocytes

Oocytes were prepared following a similar protocol as that described by Norimatsu et al. (2012). Female *Xenopus laevis* (Xenopus Express) were anesthetized by immersion in water containing tricaine (1.5 g/l) and sodium bicarbonate (0.2 g/l). The oocytes were removed through a small abdominal incision that was then closed by 4.0 nylon suture. Frogs were allowed to recover in their tanks. The follicular membranes were removed by mechanical agitation (1 − 2 h) in a Ca^2+^-free solution containing 82.5 mM NaCl, 2 mM KCl, 1 mM MgCl_2_, 5 mM HEPES (pH 7.5), and 0.2 Wünsch units/ml Liberase Blendzyme. Stage V and VI defolliculated oocytes were selected, washed, and incubated at 18 °C in a modified Barth’s solution (MBSH) containing 88 mM NaCl, 1 mM KCl, 0.82 mM MgSO_4_, 0.33 mM Ca(NO_3_)_2_, 0.41 mM CaCl_2_, 2.4 mM NaHCO_3_, 10 mM HEPES hemisodium (pH 7.5), with penicillin (100 units/ml), streptomycin (100 µg/ml), and amphotericin B (2.5 µg/ml) until injection the next day. Oocytes were coinjected with 0.1 − 10 ng of α9α10 cRNA (1:1 molar ratio, 50 nl volume) using a microinjector (Drummond Scientific). Injected oocytes were incubated at 18 °C in 12-well plates containing MBSH. Injection pipettes were pulled from filamented glass capillary tubes (Sutter Instrument) on a P-97 Flaming−Brown micropipette puller. Oocytes were used 3 − 5 days after injection.

### Electrophysiological recordings in oocytes

Individual oocytes were placed in a 200 µL RC-1Z recording chamber (Warner Instruments) and gravity-perfused with Frog Ringer’s solution (98 mM NaCl, 2 mM KCl, 1 mM MgCl_2_, 1.8 mM CaCl_2_, and 5 mM HEPES hemisodium, pH 7.4) at ∼1.5 ml/min. All solutions also contained 0.1 mg/ml BSA to reduce nonspecific adsorption of the peptide, as described by Vincler et al. (2006).

Membrane currents were recorded from oocytes with a two-electrode voltage-clamp amplifier (TEV-200; Dagan) at room temperature (∼22 °C). Electrodes had resistances of 0.5 to 2 MΩ when filled with 3 M KCl. The membrane potential was clamped at −70 mV. Data acquisition utilized an analog-to-digital converter (Digidata 1320A; Molecular Devices), and data acquisition as well as analysis was done on a Pentium-based microcomputer using pCLAMP software. Data were low-pass filtered (5 Hz cutoff) and digitized at a sampling frequency of 20 Hz.

To apply a pulse of ACh to the oocyte, the perfusion fluid was switched to one containing 10 µM ACh for 1 s. This was done at intervals of ∼6 min and has previously been shown to allow reproducible control responses without substantial desensitization (Vincler et al., 2006). To measure block by α-conopeptides, the perfusion system was stopped, the solution from around the oocyte was removed via a mechanical pipetter, and the bath was filled with 200 µl of a solution containing one of the peptides (either 1 µM Vc1.1 or 100 nM RgIA). The oocyte was incubated with the conopeptide for 5 min in the static bath. The perfusion system was then restarted with a 1 s pulse of ACh. The conopeptide dwell time was sufficiently long-lasting that the majority of α9α10 nAChR were still blocked (<2 s of wash time) when the ACh pulse arrived at the oocyte (Vincler et al., 2006). Control ACh responses prior to peptide application were exposed to the same procedure except that control Frog Ringers was used instead of peptide-containing solution. Control ACh responses were measured from the average of two preceding responses and the first response following recovery from conopeptide block (∼6 min of washout).

#### Statistics

All data are presented as mean ± SD. Two-tailed one-sample *t* tests (Excel) were used to determine significant differences (*p* < 0.05) versus zero of normally distributed data, while a Wilcoxon rank-sum analysis (IgorPro) was used to determine significant differences for data deviating from a normal distribution. The Pearson correlation test (Excel) was used to test for significant correlations between data sets.

#### Drugs and chemicals

MEM, FBS, and penicillin-streptomycin were purchased from Life Technologies. Liberase Blendzyme and collagenase were from Roche Molecular Biochemicals, and trypsin was from Worthington. α-conopeptides Vc1.1 and RgIA were synthesized as reported previously (Cartier et al., 1996; Ellison et al., 2008). All other chemicals were obtained from Sigma-Aldrich.

## Results

**Fig. 1:**
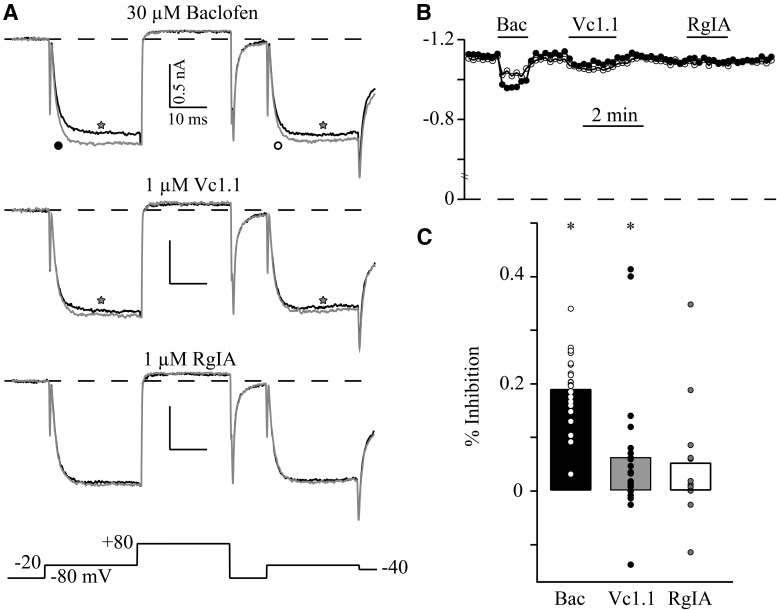
The effect of α9a10 nAChR blockers Vc1.1 and RgIA on Ca_V_ currents in rat DRG neurons. ***A***, Superimposed traces of Ba^2+^ currents from one cell in the absence (grey) and presence (black) of 30 µM baclofen, 1µM Vc1.1, and 1 µM RgIA. Voltage protocol is shown at the bottom. ***B***, The blocking time course of prepulse (filled circles) and postpulse (empty circles) current by baclofen, Vc1.1, and RgIA. ***C***, The plot shows mean percent Ca_V_ current inhibition by baclofen (*n* = 21), Vc1.1 (*n* = 21), and RgIA (*n* = 12), along with the individual data points to illustrate the large variability in responses. * indicates significant inhibition (*p* < 0.05).

Inhibition of Ca_V_ current is one mechanism that has been proposed for analgesia induced by the α-conopeptides Vc1.1 and RgIA (Callaghan et al., 2008; Cuny et al., 2012). The effects of Vc1.1 and RgIA on Ca_V_ current were studied in sensory neurons dissociated from adult rats. Since the Ca_V_ current inhibition by these conopeptides has been reported to be mediated by GABA_B_ receptors, the specific GABA_B_ receptor agonist, baclofen, was used to test for functional presence of GABA_B_ receptors by measuring Ca_V_ current inhibition (Tosetti et al., 2002). Ca_V_ current was tested using a triple-pulse voltage protocol to examine the voltage dependence of inhibition (Elmslie et al., 1990), which results from transient disruption of G protein βγ subunits binding to Ca_V_2 channels (Ikeda, 1996). Thirty micromolar baclofen significantly inhibited prepulse currents by 19.2 ± 6.8% (mean ± SD, *n* = 21; Fig. 1). As expected, this inhibition was voltage-dependent since the postpulse current was inhibited by only 12.0 ± 5.5%, which was significantly smaller than the prepulse inhibition (*p* = 0.005). Ca_V_ current in 20/21 (95%) sensory neurons was inhibited by baclofen.

The effect of Vc1.1 (1 µM) and RgIA (1 µM) on Ca_V_ current differed from that of baclofen (Fig. 1). While inhibition was observed in some neurons by each conopeptide, the overall effect was a small but significant prepulse current inhibition by Vc1.1 (6.5 ± 12.7%, *n* = 21, *p* = 0.003; Fig. 1). This inhibition was not voltage-dependent since the postpulse inhibition was 6.2 ± 12.5%. There was no significant inhibition by RgIA of either the prepulse (5.5 ± 11.7%, *n* = 12, *p* = 0.077, n.s.) or postpulse current (5.3 ± 10.7%).

**Fig. 2:**
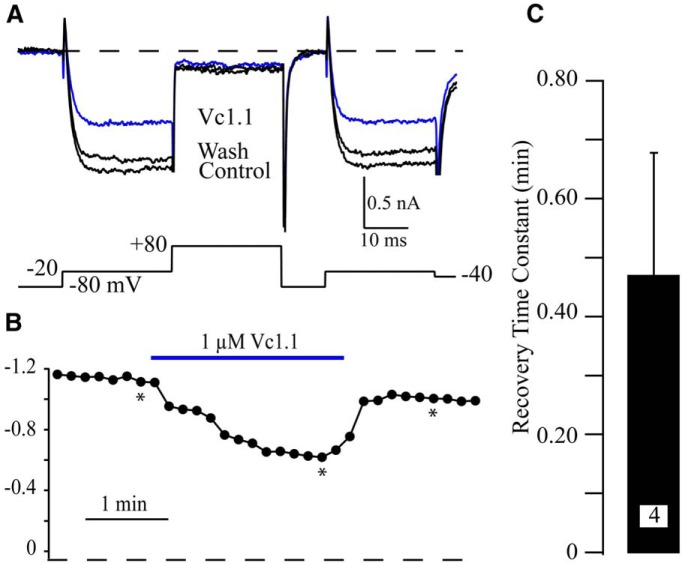
Rapid recovery from Vc1.1-induced inhibition. ***A***, Example traces from a neuron with a 40% inhibition of Ca_V_ current induced by 1 µM Vc1.1 (blue trace). Note the almost full recovery from inhibition in the washout trace (Wash). ***B***, The time course of inhibition by Vc1.1. The asterisks indicate the traces used in ***A***. ***C***, The average time constant (τ) for recovery from block by 1 µM Vc1.1 from the four neurons with inhibition >10%. Recovery τ was determined by fitting the Vc1.1 washout time course using a single exponential equation.

While the effect of Vc1.1 and RgIA was on average small or insignificant, there were a few neurons that responded with inhibitions >10%. This includes 4/21 (19%) neurons tested with Vc1.1 and 2/12 (17%) neurons tested with RgIA. This contrasts with the previous report showing that Ca_V_ current in ∼75% of sensory neurons was inhibited by 100 nM Vc1.1 (Callaghan et al., 2008). We wanted to further investigate the peptide-induced inhibition to determine if the properties were similar to those reported previously (Callaghan et al., 2008). It was previously reported that Ca_V_ current block by Vc1.1 was irreversible, but we found that the block by Vc1.1 was readily reversible with an average recovery τ = 0.5 ± 0.2 min (*n* = 4; Fig. 2). Thus, this inhibition appears to be distinct from that previously reported (Callaghan et al., 2008).

**Fig. 3:**
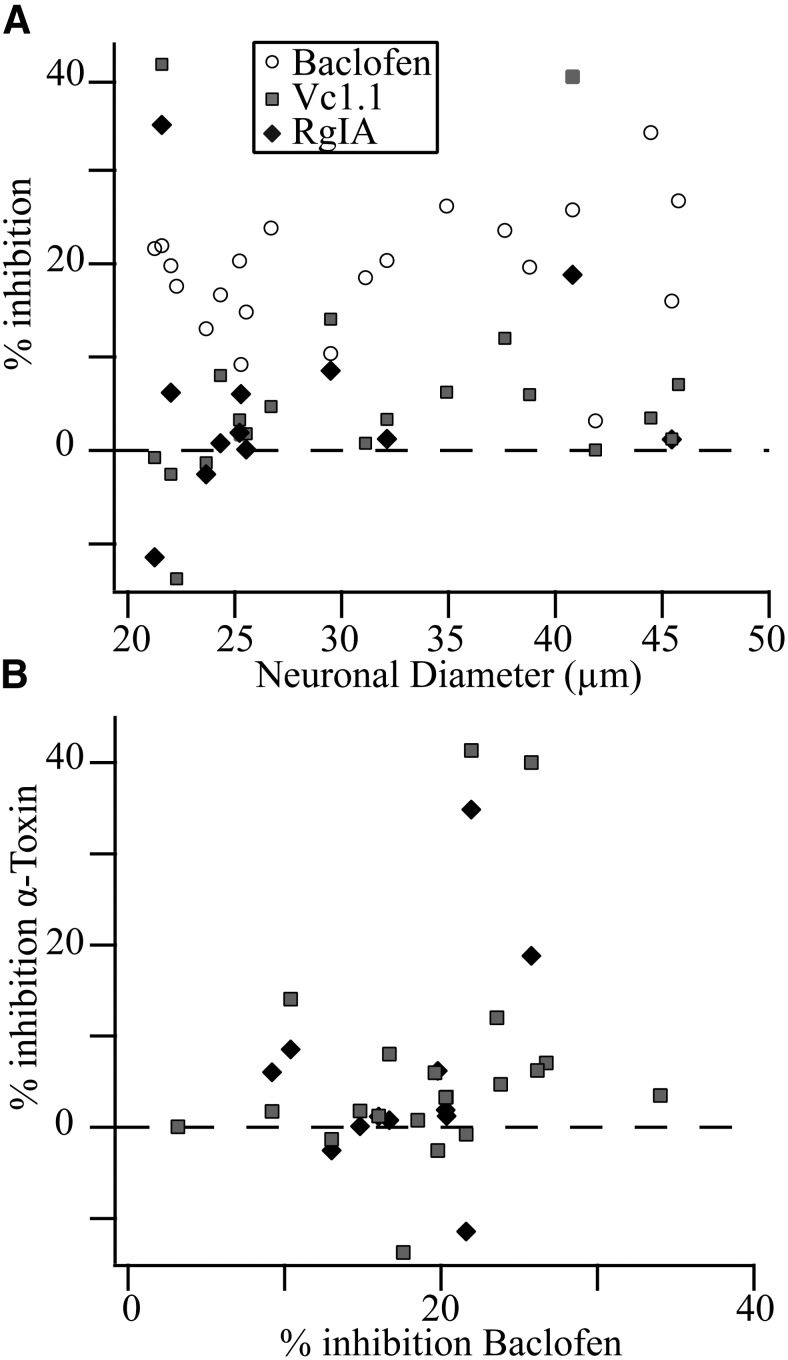
Inhibition by α-conopeptides Vc1.1 or RgIA does not correlate with inhibition by the GABA_B_ agonist baclofen. ***A***, The percent inhibition of prepulse Ca_V_ current by 30 µM baclofen, 1 µM Vc1.1, or 1 µM RgIA is plotted versus cell diameter (calculated as described in Materials and Methods). ***B***, The percent inhibition of prepulse Ca_V_ current by α-conopeptides Vc1.1 or RgIA is plotted versus percent inhibition by baclofen, and no correlation was observed.

Another question was if a particular group of neurons exhibited conopeptide sensitivity. One possibility is that the sensitive neurons were nociceptors, which would predict that the somal diameter of these neurons would be smaller (<35 µm) than the unresponsive, non-nociceptive neurons (Djouhri et al., 2003). This possibility was investigated by plotting the somal diameter versus percentage prepulse inhibition (Fig. 3*A*). Against this prediction, the “responsive” neurons spanned the size range with large neurons (>35 µm) just as likely (*n* = 2) to respond to the conopeptides than small neurons (<30 µm, *n* = 2; Fig. 3*A*). Thus, the evidence suggests that the conopeptide-induced Ca_V_ current inhibition is not a marker for nociceptive sensory neurons.

Surprisingly, the baclofen-induced prepulse inhibition did not appear to correlate with that induced by Vc1.1 (Fig. 3*A*), as expected if GABA_B_ receptors mediate Vc1.1-induced inhibition. This relationship was more fully investigated by plotting the Vc1.1-induced Ca_V_ current inhibition versus that induced by baclofen (Fig 3*B*). Calculation of the Pearson correlation yielded *R* = 0.27 (n.s.). For completeness, the RgIA data are also plotted (Fig. 3*B*) and the Pearson correlation was *R* = 0.29 (n.s.). Thus, no correlation was found between the responses induced by either Vc1.1 or RgIA versus baclofen. Notably, the neuron with the largest baclofen response (34%) showed only a 3.5% Ca_V_ current inhibition by Vc1.1 (Fig. 3*B*). The neuron with the largest Vc1.1 (41%) and RgIA (35%) showed a 22% inhibition by baclofen. However, seven other neurons with baclofen responses ranging from 20 − 24% responded to Vc1.1 with an average 4.3 ± 6.6% inhibition of Ca_V_ current, while the four neurons also tested with RgIA responded with a 0.0 ± 7.6% effect. It appears that GABA_B_ receptors do not mediate the small Ca_V_ current inhibition induced by Vc1.1.

Interestingly, there was a significant correlation found between the Ca_V_ inhibitions induced by Vc1.1 and RgIA (*R* = 0.88, *p* < 0.05). This result suggests a common inhibitory mechanism for both Vc1.1 and RgIA, but as mentioned above, we could find no evidence that GABA_B_ receptors mediate this Ca_V_ current inhibition.

**Fig. 4:**
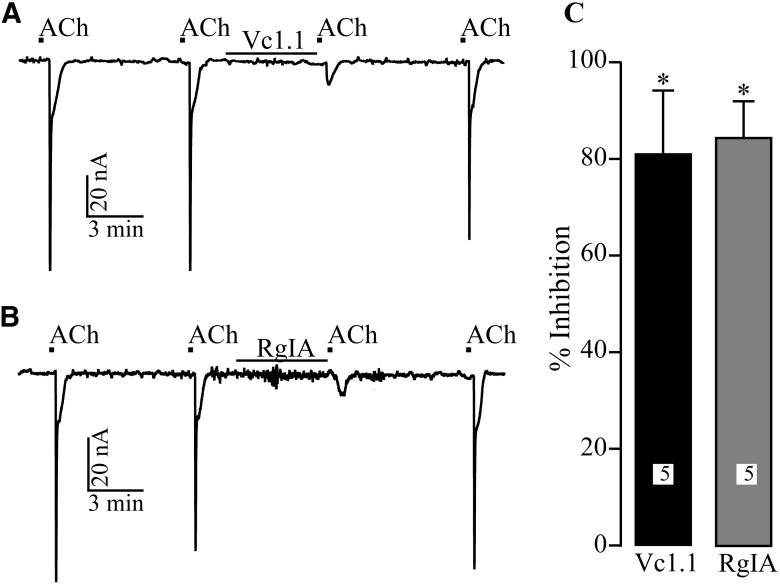
Vc1.1 and RgIA potently block α9α10 nAChRs expressed in *X. laevis* oocytes. ACh-induced currents were measured from voltage-clamped oocytes as described in Materials and Methods. ***A***, ***B***, Representative traces of ACh-induced currents in the presence and absence of Vc1.1 or RgIA, respectively. ***C***, The mean (±SD) inhibition of ACh-induced peak current amplitude by α-conopeptides Vc1.1 and RgIA. The numbers in bars reflects numbers of cells tested. * indicates significant inhibition (*p* < 0.05).

As a positive control, *Xenopus* oocytes expressing rat α9α10 nAChRs were exposed to Vc1.1 or RgIA to ensure the conopeptides were functional. ACh-induced currents were recorded using the two-electrode voltage-clamp method. Application of ACh (10 µM) was limited to 1 s in duration once every 5 − 6 min. This protocol yielded stable ACh-induced currents (Fig. 4) with average amplitude of 768 ± 275 nA. Consistent with other reports (Vincler et al., 2006), Vc1.1 (1 µM) and RgIA (100 nM) significantly blocked ACh-induced current by 85 ± 13% and 81 ± 8%, respectively (Fig. 4). Thus, these conopeptides block α9α10 nAChRs as expected, yet fail to substantially inhibit Ca_V_ current in rat sensory neurons.

## Discussion

In previous work, the maximum inhibition of Ca_V_ current in sensory neurons isolated from either rat or mouse DRG was 40 − 50% by 1 µM of either Vc1.1 or RgIA (Callaghan et al., 2008; Callaghan and Adams, 2010). The mechanism of this inhibition was concluded to be mediated by GABA_B_ receptor activation. We also utilized 1 µM Vc1.1 and RgIA, but found on average only small (7%, Vc1.1) and insignificant (RgIA) effects on Ca_V_ current in rat sensory neurons. The role of GABA_B_ receptors was also assessed, but there was no correlation between the magnitudes of baclofen- and Vc1.1-induced inhibitions. These same α-conopeptides that minimally affected Ca_V_ current strongly (>80%) inhibited α9α10 nAChRs expressed in *Xenopus* oocytes, which demonstrates the expected potency of these α9α10 nAChR antagonists. While our results fail to reproduce results reported in some previous publications (Callaghan et al., 2008; Callaghan and Adams, 2010), they do support other publications showing that RgIA and Vc1.1 do not activate GABA_B_ receptors expressed in *Xenopus* oocytes (McIntosh et al., 2009) and showing that Vc1.1 does not affect excitatory neurotransmitter release from sensory nerve terminals that express GABA_B_ receptors (Napier et al., 2012).

### Different effects

While the overall inhibition was small, we found that these conopeptides could inhibit Ca_V_ current (>10%) in a minority of sensory neurons (<20%). This fraction of sensitive neurons is much smaller than that previously reported (75%) (Callaghan et al., 2008). Other differences include a relative fast recovery from block versus irreversible block, and the apparent lack of GABA_B_ receptor involvement (Callaghan et al., 2008). We were unable to identify a single neuronal group as conopeptide sensitive, since a few small (<30 µm), medium, and large (>40 µm) diameter neurons were found to be sensitive, while other neurons within the same size range were insensitive. As a result, it seems unlikely that nociceptors define the conopeptide-sensitive population.

We have no data to explain why we cannot reproduce the previously published Ca_V_ current inhibitions (Callaghan et al., 2008; Callaghan and Adams, 2010). However, we can exclude some possibilities. First, the α-conopeptides used here were potent inhibitors of α9α10 nAChRs, which demonstrated that they were functional peptides. Second, the previous publications demonstrated that N-type Ca_V_ channels were the Ca_V_ channel type inhibited by Vc1.1 and RgIA (Callaghan et al., 2008), and we have demonstrated N-type channels comprise approximately half of the total Ca_V_ current in rat sensory neurons (Ramachandra et al., 2012). Thus, the absence of the target channel cannot explain the differences. Finally, the GABA_B_ receptors were functional in these neurons since the specific agonist, baclofen, inhibited Ca_V_ current in 95% of neurons tested, which confirms the presence of the putative receptor that mediates the Ca_V_ current inhibition induced by these α-conopeptides.

Differences among species have been proposed as a possible reason for differing results. McIntosh et al. (2009) demonstrated that both Vc1.1 and RgIA failed to block binding of [^3^H]CGP-54626, a specific competitive antagonist, to human GABA_B_ receptors, and suggested that the human receptors were not a target for these conopeptides. However, Ca_V_ current inhibition by Vc1.1 and RgIA has been shown in both rat and mouse sensory neurons (Callaghan et al., 2008; Callaghan and Adams, 2010), and our results from rat sensory neurons fit well with the human data. Thus, species differences are unlikely to explain these differences.

The sources of the conopeptide are different, but it is not clear how that would explain the different results. These peptides are synthesized by manual solid-phase synthesis. Disulfide bond formation is by directed synthesis and/or verified by NMR analysis. The peptides are purified in a similar manner between labs with reversed-phase high-performance liquid chromatography using trifluoracetic acid and acetonitrile buffer systems.

### Analgesic mechanisms

Many experiments have demonstrated the analgesic properties of α-conopeptides that block α9α10 nAChR, including Vc1.1 and RgIA (Satkunanathan et al., 2005; Vincler et al., 2006; Napier et al., 2012; Di Cesare Mannelli et al., 2014). However, post-translational modifications of Vc1.1 that preserved α9α10 nAChR block eliminated the analgesic effects (Nevin et al., 2007). This suggested that the analgesic effect of Vc1.1 did not result from α9α10 nAChR block. Interestingly, the effect of Vc1.1 to inhibit Ca_V_ current was lost by these same post-translational modifications, which supported Ca_V_ current inhibition as an analgesic mechanism for Vc1.1 (Callaghan et al., 2008). However, the pharamacokinetic properties of this analog were not investigated, leaving open the possibility that the compound did not reach its *in vivo* target in adequate concentration. In the present study, we found, on average, little to no Ca_V_ current inhibition by Vc1.1 or RgIA in sensory neurons, in contrast to prior reports. These overall findings agree with a recent publication that demonstrated no inhibition of EPSPs in secondary sensory neurons in the dorsal horn by Vc1.1, even though the EPSP was strongly inhibited by baclofen (Napier et al., 2012).

The analgesic mechanism of the ω-conopeptide, ziconitide, involves the direct block of presynpatic Ca_V_2.2 channels to decrease glutamate release and the resulting EPSP in secondary nociceptors (Elmslie, 2004), which blocks pain transmission between primary nociceptors and second-order neurons in the dorsal horn of the spinal cord (Vanegas and Schaible, 2000; Elmslie, 2004). RgIA and Vc1.1 are unlikely to cross the blood−brain barrier and reach spinal neuron synapses, which further suggests that RgIA and Vc1.1 induced analgesia may not involve Ca_V_ channels. In addition, the highly selective N-type Ca_V_ antagonist, ziconotide, did not decrease neuropathic pain when given peripherally by intravenous injection (Chaplan et al., 1994). This FDA-approved drug must be delivered by intrathecal administration for therapeutic effect (Sanford, 2013). Furthermore, N-type channel expression has been reported to be reduced in peripheral sensory neurons after nerve injury (McCallum et al., 2011). Together, these findings suggest non-Ca_V_ channel mechanisms are important for RgIA- and Vc1.1-induced analgesia.

Recent work has demonstrated a possible role of α9 nAChR in pain, since mechanical hyperalgesia was reduced in α9 nAChR knockout mice following chronic nerve constriction and in an inflammatory pain model (Mohammadi and Christie, 2014). In addition, a major effect of RgIA appears to be on the glial/immunological response to chronic nerve injury to prevent pathological changes within the nervous system that are thought to result in neuropathic pain (Di Cesare Mannelli et al., 2014). There are also small molecule antagonists of α9α10 nAChRs. These compounds have also been shown to be analgesic, lending further support for the importance of this mechanism (Holtman et al., 2011; Zheng et al., 2011; Wala et al., 2012). While the present study does not allow us to identify the mechanism by which Vc1.1 or RgIA produce analgesia, our findings do not support a role for Ca_V_ channel inhibition in sensory neurons as one of those mechanisms.

## Synthesis

The decision was a result of the Reviewing Editor Douglas Bayliss and the peer reviewers coming together and discussing their recommendations until a consensus was reached. A fact-based synthesis statement explaining their decision and outlining what is needed to prepare a revision is listed below. The following reviewers agreed to reveal their identity: Stephen Ikeda and Diane Lipscombe

This paper addresses the mechanism of action of two analgesic alpha-conopeptides, specifically testing a prominent hypothesis that they act on primary sensory neurons by activating GABA-B receptors to cause inhibition of N-type calcium channels. As evident in Referees' comments, the convincing negative data provide an important counterpoint to that prevailing hypothesis, which should stimulate a re-examination of how these compounds produce their analgesic actions. As such, it will be a valuable contribution to the field.

The major concerns for both Reviewers center on the analysis and presentation of the data. Specifically, DRG neurons are heterogenous and there is substantial cell-to-cell variability in effects of the conopeptides on calcium current. This variability should be better represented in the figures, and more explicitly acknowledged in the description and discussion of results. Also, more appropriate statistical analyses of the data should be undertaken (non-parametric analysis for Fig. 1c, formal correlational analysis in Fig. 3), and some additional consideration of the correlation between effects of the two conopeptides should be provided.

Details regarding these particular recommendations, along with additional suggestions for improvement may be found in the reviewer comments.

### Reviewer 1

This manuscript addresses an interesting controversy regarding the mode of action of the alpha-conopeptides Vc1.1 and RgIA. Previous work, mostly from the David Adams group, identify the peptides as antagonist at certain nAChR subtypes and, somewhat surprisingly, as agonist at GABAb receptors. At present, the Adams group concludes that the peptides act via a GABAbR to inhibit N-type calcium channels in DRG neurons (terminals) and hence act as analgesics. The initial assumption that nAChR are involved in the analgesia has been discarded. Although originally put forth as a simple orthosteric agonist at GABAbR, I believe the story is evolving to a much more complex mechanism (some weird form of allostery). Taken together with negative or contradictory results from a couple of groups, the mechanism of how these peptides work remains unclear. Hence, the data presented in this manuscript are of interest and serve to push the field forward.

The majority of work in this manuscript was done on dissociated rat DRG neurons using standard whole-cell patch clamp recordings. The last experiment is done in Xenopus oocytes expressing nAChR. The overall result is negative. The conopeptides seem to have minimal effects on calcium channels in DRG neurons and in the few neurons that do respond, no correlation with the size of the baclofen response was seen. The Xenopus experiments show the toxin were active at nAChR (figure 4) and serves as a type of positive control for the negative calcium channel data. The authors do not provide an explanation for the discrepancy between their data and the Adams data although a few ideas are put forward.

Overall I find the work convincing but have 2 major points that I would like to see addressed.

Major points:

1) Statistical analyses and data presentation could be improved/clarified.
  - The bar graph in figure 1C would be more informative if the individual data points were also shown. DRG neurons are notorious for their heterogeneity and the distribution of data points would be informative. Also, the authors should indicate the type of t-test used. I assume in this case a two-tailed one-sample t-test (vs. 0).
    Normality tests (D'Agostino & Pearson omnibus normality test in this case - Prism) suggest that toxin inhibition (both) were not normally distributed (whereas diameter and baclofen was). Hence, t-tests in general probably aren't a good idea. However, I ran a Wilcoxon (non-parametric) and the results were unchanged. It is important the authors show the individual data points instead of the bar graphs alone.
  - For Figure 3, the lack of correlation could be backed up by statistical analyses. The authors should formally analyze these correlation data.
    I ran the standard Pearson's correlation on the data and it supports the author's contention. I should mention that there is significant correlation (P=1.68e-04) between the Vc1.1 vs RgIA inhibition which is somewhat interesting. The authors might mention this. The fact that the Vc1.1 and RgIA inhibitions are correlated would suggest that a specific target is being affected by the toxins but this is not the GABAbR.
  - The analyses discussed in lines 204-208 aren't very useful and should be considered for deletion. First, I ran this analysis and didn't come up with a significant difference for either group (Vc1.1 <30 um or > 30 um). Second, comparing significance between groups is not in itself significant (refs below).
    Nieuwenhuis S, Forstmann BU, Wagenmakers E-J (2011) Erroneous analyses of interactions in neuroscience: a problem of significance. Nat Neurosci 14:1105-1107.
    Gelman A, Stern H (2006) The Difference Between "Significant" and 'Not Significant' is not Itself Statistically Significant. The American Statistician 60:328-331.
2) Source of peptides. A potential difference between the Adams work and this work might be the peptides themselves. The authors should discuss this point if possible. What is the purity of peptides and are there issues with things like disulfide bound formation. Is it possible that contaminants (e.g. salts) might account for some of the differences?

Minor points

1) line 103: degree of series resistance compensation should be mentioned.
2) line 141: units (Mohms) missing.
3) Figure 2A. The current traces show a sizable inward current at +80 mV. Normally there is little current at this potential or it is slightly outward (e.g., Figure 1A). What is the explanation for this?

### Reviewer 2

The purpose of this study was to assess the inhibitor efficacy of two peptide toxins Vc1.1 and RgIA on CaV2.2 channels expressed in sensory neurons. The significance of the study is as follows: Intramuscular injection of Vc1.1 and RgIA are analgesic in response to mechanical stimulation in peripheral nerve injury models that induce chronic pain. These conus peptide toxins inhibit neuronal nicotinic receptors of the a9a10 subtype - a finding that was reproduced in this study. However, the authors find little support for the proposal that Vc1.1 and RgIA conotoxins also inhibit voltage-gated CaV2.2 channels via action on GABAb receptors - a conclusion from a series of publications by DJ Adams and colleagues. There are clearly some important findings in this manuscript that are important to the field. The authors show that Vc1.1 and RgIA have highly variable actions on CaV currents in a heterogenous population of sensory neurons, with some non-responders, and a few relatively large responders. They nicely show scatter plots but in their conclusions compressing their highly variable data into a single point. Their results are clearly different from what has been published by Adams and colleagues and this is important. However, as written, their conclusions over simplify the data shown.

1. The authors conclude that Vc1.1 and RgIA on average are at best weak inhibitors of CaV currents in sensory neurons. However, more importantly, they show that the efficacy of these toxins varies greatly across a heterogenous population of sensory neurons. CaV currents in some cells are non responsive to the toxins, while there is greater inhibition of CaV currents in other cells. In 25% of neurons (5/21), Vc1.1 inhibited CaV currents by more than 10%. To illustrate this variability, rather than show the average values +/- SD in the form of histograms, it would be more informative if the authors show the scatter in the data by illustrating individual points. The authors have not determined the source of the variability - this does not necessarily matter - but they should discuss this as a variable.
2. Related to the above. In several places in the manuscript including the title the authors suggest these toxins have minimal or no effect of CaV currents. As noted in the summary above, I don't think this accurately reflects what the authors are showing. They show highly variable inhibition, with some examples of no inhibition, some with weak inhibition, and a few (25%) relatively strong inhibition of CaV currents in sensory neurons.
3. The authors suggest that inhibition of CaV by Vc1.1 is significant but that by RgIA is not significant (no p values were show for this). The data sets for both toxins look very similar and indeed they are overlapping. I think the use of p < 0.05 (in testing whether inhibition by each toxin is greater than zero) is not useful and, given the high variability in toxin action, the data set would have to be much larger for each toxin to test whether the two toxins had differential effects on CaV currents in this heterogenous population of sensory neurons.
4. The authors should use absolute p values to replace p < 0.05
5. The authors find that neurons of smaller diameter - nociceptors - are less sensitive to Vc1.1 than those with diameters larger than 30 um. But both Aδ and C fibers carry nociceptive signals and are associated with small to medium diameter neurons. Vc1.1 is effective against noxious mechanical stimuli perhaps indicating that it might be effective on medium-size neurons.
6. P.14 line 283 "In the present study, little or no CaV current inhibition by Vc1.1 or RgIA in sensory neurons was found, in contrast......" This sentence does not accurately represent what the authors have found. They find highly variable effects of both toxins on CaV currents in undefined sensory neurons.
7. P.11 line 204-208. The value added to having both these sentences is not clear.
8. P.14 line 262-271 Unless I am missing something, this section of the discussion appears to be addressing a different aspect of the work on Vc1.1 and other toxins including AuIB and inhibition of neuronal nAChRs published by Adams and colleague that are not addressed directly by the authors. It seems tangential and distracting.
9. P.10 line 190-196 The authors initially talk about RgIA "We wanted to further investigate this inhibition to determine if......" and then switch to Vc1.1 in the next sentence?
